# Using prior information to enhance microwave tomography images in bone health assessment

**DOI:** 10.1186/s12938-021-00966-5

**Published:** 2022-02-02

**Authors:** Mohanad Alkhodari, Amer Zakaria, Nasser Qaddoumi

**Affiliations:** 1 Department of Electrical Engineering, American University of Sharjah, Sharjah, United Arab Emirates; 2 Department of Biomedical Engineering, Khalifa University, Abu Dhabi, United Arab Emirates

**Keywords:** Microwave tomography, Two-dimensional imaging, Finite-element method, Contrast source inversion, Prior information, K-means clustering, Bone degradation, Bone health assessment, Wearable system

## Abstract

**Background:**

Osteoporosis is the major cause of bone weakness and fragility in more than 10 million people in the United States. This disease causes bone fractures in the hip or spine, which result in increasing the risk of disabilities or even death. The current gold standard in osteoporosis diagnostics, X-ray, although reliable, it uses ionizing radiations that makes it unfeasible for early and continuous monitoring applications. Recently, microwave tomography (MWT) has been emerging as a biomedical imaging modality that utilizes non-ionizing electromagnetic signals to screen bones’ electrical properties. These properties are highly correlated to bones’ density, which makes MWT to be an effective and safe alternative for frequent testing in osteoporosis diagnostics.

**Results:**

Both the conventional and wearable simulated systems were successful in localizing the tibia and fibula bones in the enhanced MWT images. Furthermore, structure extraction of the leg’s model from the blind MWT images had a minimal error compared to the original one (L2-norm: 15.60%). Under five sequentially incremental bone volume fraction (BVF) scenarios simulating bones’ treatment procedure, bones were detected successfully and their densities were found to be inversely proportional to the real part of the relative permittivity values.

**Conclusions:**

This study paves the way towards implementing a safe and user-friendly MWT system that can be wearable to monitor bone degradation or treatment for osteoporosis cases.

**Methods:**

An anatomically realistic finite-element (FE) model representing the human leg was initially generated and filled with corresponding tissues’ (skin, fat, muscles, and bones) dielectric properties. Then, numerically, the forward and inverse MWT problems were solved within the framework of the finite-element method-contrast source inversion algorithm (FEM-CSI). Furthermore, image reconstruction enhancements were investigated by utilizing prior information about different tissues as an inhomogeneous background as well as by adjusting the imaging domain and antennas locations based on the prior structural information. In addition, the utilization of a medically approved matching medium that can be used in wearable applications, namely an ultrasound gel, was suggested. Additionally, an approach based on k-means clustering was developed to extract the prior structural information from blind reconstructions. Finally, the enhanced images were used to monitor variations in BVF.

**Graphical Abstract:**

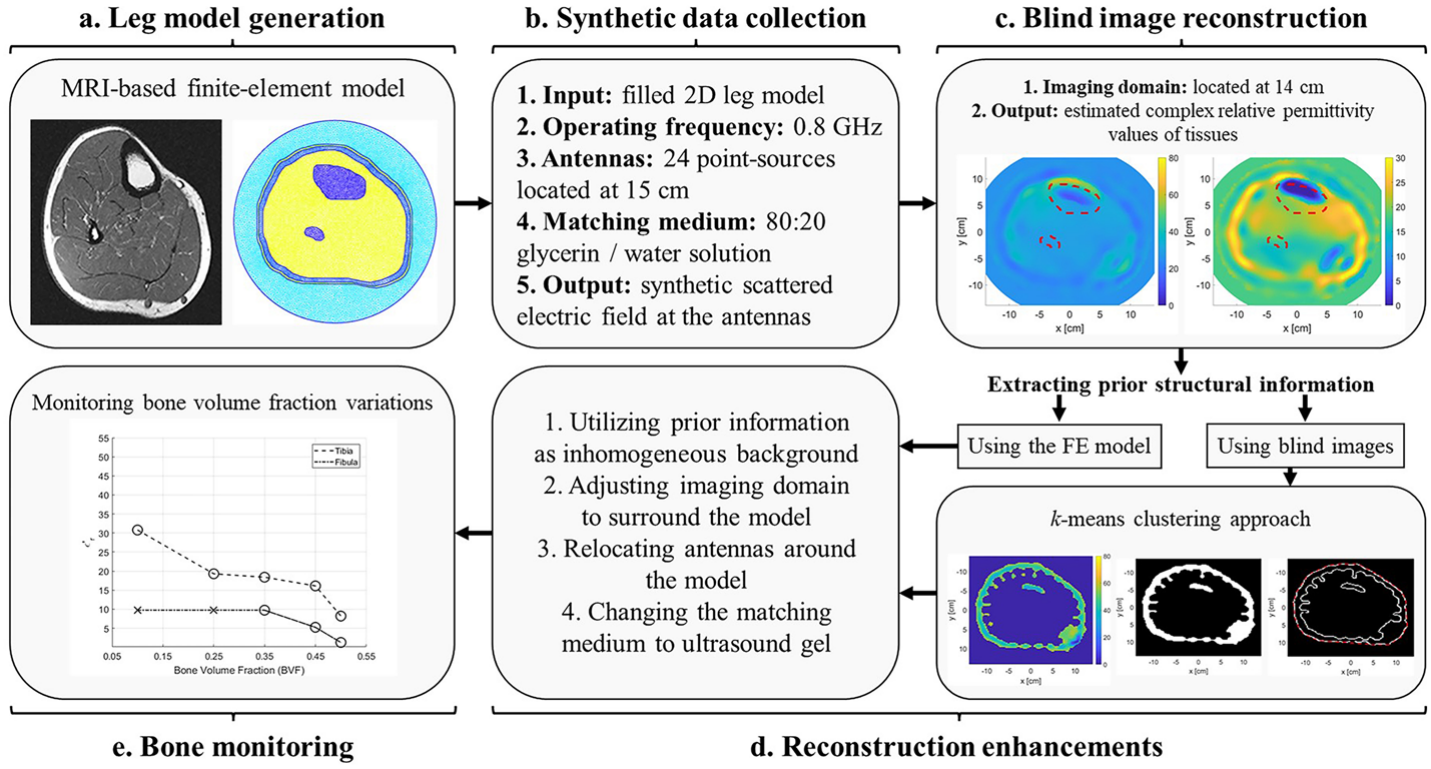

## Introduction

Microwave tomography (MWT) has been emerging as a promising imaging technology in various medical fields for the past 3 decades [1–3]. Some of the biomedical MWT applications include breast cancer diagnostics [4–6], brain stroke early detection [7–9], extremities imaging [10, 11], and lung cancer detection [12, 13]. In comparison to other medical imaging modalities, MWT offers several advantages such as its use of low-power non-ionizing electromagnetic signals, its relatively lower cost in comparison to X-ray and magnetic resonant imaging (MRI) [2], and its ability to estimate two distinct electrical properties of an object-of-interest (OI), which are the OI’s relative permittivity (dielectric constant) and effective conductivity.

In biomedical microwave tomography, an antenna array surrounds a human organ (the object-of-interest (OI)) located in the middle of an imaging chamber filled with a matching medium. Each element in the antenna array acts as a transmitter and receiver interchangeably. During measurements, each antenna radiates the OI simultaneously with an electromagnetic signal. For each transmitting antenna, the other antennas operate as receivers and measure the fields scattered around the OI. The collected data are used as inputs for an optimization algorithm to produce two-dimensional (2D) tomographic images of the OI; the images depict the algorithm’s estimation for the location and electrical properties of the bulk tissues within the human organ. Since, mathematically, MWT involves solving a non-linear ill-posed inverse scattering problem, specialized optimization algorithms in conjunction with regularization techniques are utilized. Examples of such algorithms include the contrast source inversion [14], Gauss–Newton inversion [15], and level-set methods [16].

A potential application for MWT in medical diagnosis is the monitoring of bone health, which is crucial with the increase in bone diseases such as osteoporosis [17, 18]. According to the National Osteoporosis Foundation (NOF), more than 10 million people in the United States are affected by osteoporosis, with an additional 33.6 million suffering from low bone density of the hip [19]. One of the main causes of osteoporosis is Vitamin D deficiency [20, 21]; Vitamin D enables the body to absorb calcium, which is necessary for bones to maintain their health and strength. As reported by the International Osteoporosis Foundation (IOF), the United Arab Emirates (where the presented research is conducted) is one of the top counties with both degeneration cases: Osteoporosis and Vitamin D deficiency. It is estimated that 78% of the UAE’s population suffers from Vitamin D deficiency [22]. This is due to several factors such as genetics, lifestyle, low activity, obesity, and cultural dress codes. Osteoporosis can affect both types of bones; the trabecular and cortical bones [23, 24]; however, cortical bones are more solid and stiff, which allow for further bone strength and density degradation investigations due to osteoporosis.

Currently, the golden standards considered for the clinical assessment of bone mass and strength are the dual-energy X-ray absorptiometry (DXA) and the quantitative computed tomography (QCT). In these systems, examinations are done for the anatomical structures of the body such as the hip, the spine, and the wrist, where the denser cortical bones are more dominant than the spongy trabecular bones [17]. In current gold standard techniques, osteoporosis can be monitored from both trabecular and cortical bone types [24]. A major drawback of these systems is their excessive use of ionizing X-ray radiation emitted to human bodies, which may lead to health challenges for long-term monitoring. In addition, the overall cost of these machines is considered extremely high [17, 25]; with the use of MWT, the drawbacks of DXA and QCT can be resolved. Since MWT uses non-ionizing radiation, monitoring the bones’ health on regular basis can be performed safely, which is important in evaluating the efficacy of long-term treatments of bone osteoporosis. It should be noted here that MWT images the bulk electrical properties of an object, which is different than the thorough image reconstructions from X-ray imaging; this might be misperceived as a disadvantage in MWT.

To assess bone mass, the bone volume fraction (BVF) metric is commonly used to represent the strength and quality of bone in patients. The BVF parameter represents the volume of the mineralized bone per unit volume of the sample [26]. Moreover, BVF decreases as the bone density decreases, which represents deterioration in the bone health. Furthermore, it has been shown experimentally by [27, 28] that BVF is highly correlated with the electrical properties of the bones. More specifically, BVF is inversely proportional to the bones’ dielectric constant and effective conductivity. Therefore, using MWT to estimate the electrical properties of the bones, bone health can be monitored.

**Fig. 1:**
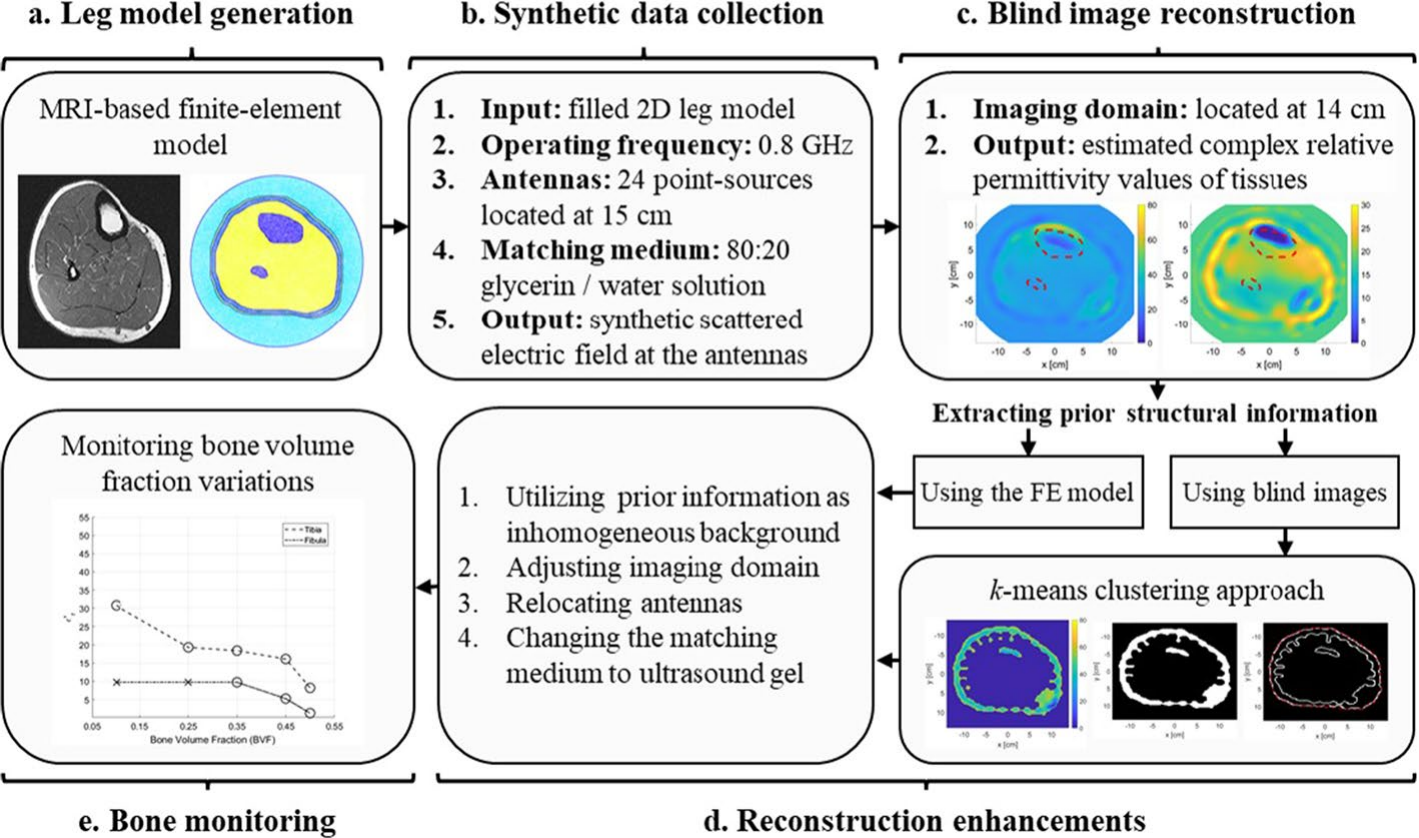
The structure of the proposed numerical microwave tomography (MWT) approach for bone analysis: **a** numerical modeling of a cross-sectional MRI-derived image to generate an anatomically realistic finite-element (FE) model; **b** calculating the synthetic data (scattered electric field values) at the antennas; **c** an initial image of the object-of-interest (OI) reconstructed blindly; **d** several image enhancements techniques were used to better enhance bones reconstruction; **e** bone monitoring following an expert-eye approach to detect variations in bone volume fraction (BVF) as variations in the corresponding dielectric properties

The current state-of-art in using MWT for bone imaging focuses on extracting the electrical properties of the calcaneus, which is also called the heel bone [11, 29–31]. In this paper, quantitative microwave imaging is performed across the lower midsection of the leg to estimate the electrical properties of the tibia and/or fibula bones; the tibia is the bigger bone located in the shaft of the lower leg, while the fibula is the smaller bone. This section of the leg is chosen, because here the bones consist mostly of cortical bones, unlike the proximal and distal parts of both bones that are comprised of mostly trabecular (spongy) tissues [32]. In studying the effect of bone degeneration and treatment processes, it is important to target the cortical bones as they are more denser and contains less fluids in comparison to the trabecular bones. In addition, the midsection of the lower leg was selected due to its accessibility, with the ability to position the antenna array of an MWT system around it easily. Furthermore, the objectives of the quantitative MWT system herein would be to locate the bone(s) within the leg and estimate its (their) electrical properties. Although such system may require more computations, it provides extra information about the OI in terms of shape, location, as well as permittivity values compared to a qualitative system that determines only the shape and location [2]. By monitoring changes in the bone’s permittivity values using a quantitative MWT system, the efficacy of bone density degradation treatments can be assessed.

In this paper, a numerical feasibility study (Fig. 1) is conducted towards the design of a wearable microwave tomography system to monitor changes in bone density within a human leg as a result of treatment. Several research groups [33–35] have developed wearable systems for various biomedical applications. In this study, we propose, through numerical simulations, different approaches towards the design of a wearable system for bone imaging. The study was carried out using an anatomically realistic MRI-derived numerical model of human leg cross-section. Using the model, synthetic data are generated using an in-house 2D finite-element method (FEM) solver [36, 37] with the transverse magnetic (TM) approximation; due to the nature of the leg cylindrical structure, this approximation is valid. The synthetic data were then inverted using the finite-element contrast source inversion (FEM-CSI) algorithm with multiplicative regularization [38, 39]. The use of FEM with CSI offers several advantages that are utilized to improve the quality of bone reconstructions. These advantages include the flexibility of choosing an arbitrary shape imaging domain for the inversion algorithm, the ability of varying the antennas’ locations, and the capability of incorporating inhomogeneous backgrounds.

It should be noted that preliminary investigations related to this work have been presented in [40–43]. In our previous work, we have utilized the blind reconstructions to investigate the detection of bone density variation in MWT; blind reconstructions are those obtained from FEM-CSI algorithm without incorporating any prior information. Furthermore, we have discussed the consequences of utilizing various image enhancement techniques in MWT. In the work presented herein, we extend these findings by applying image enhancement techniques directly on the blind reconstructions to better visualize bones automatically without the need of any additional imaging modalities. These image enhancement techniques include incorporating prior information about the leg to improve the inversion algorithm output. This prior information is obtained from the blind reconstructions. The prior information includes the location of the leg’s outer contour and information about the bulk tissues within the leg. The prior information can be easily incorporated because of the use of FEM-CSI.

In real-life scenarios, patients would perform a single imaging procedure, while the inversion algorithm will run multiple times. In the first run, blind reconstructions are obtained. Next, prior information is extracted from the blind reconstructions. This is followed by a second run for the inversion algorithm, where the obtained prior information is used to enhance the inversion algorithm output.

In the first part of the study herein, the prior information are obtained using the MRI-based leg models, to prove the concept of image enhancement. In the second part of the study, an algorithm based on k-means clustering is developed to extract the prior information from the blind inversion results.

## Results

### Imaging the anatomically realistic leg model

**Fig. 2:**
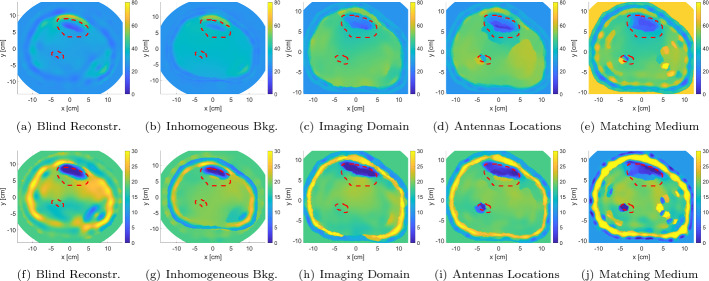
The real (top row) and imaginary (bottom row) relative complex permittivity results for the leg model: **a**, **f** blind reconstructions; **b**, **g** incorporating inhomogeneous background; **c**, **h** changing the imaging domain; **d**, **i** relocating the antennas; **e**, **j** changing the matching medium. The red-dotted lines indicate the bones’ actual locations

We have performed the imaging procedure on the developed anatomically realistic leg model (discussed in Sect. 5.1.1) through solving the forward and inverse problems of MWT. In the forward problem, the relative complex permittivity values of each tissue layer within the leg model were specified and used to calculated the scattered field on every antenna (total of 24 antennas). This scattered field was used as an input to the optimization algorithm (FEM-CSI, detailed in Sect. 5.2) to estimate the relative complex values of tissues. The iterative algorithm outputs a color map that represents the reconstruction of the relative complex permittivity values of the leg’s 2D model. This was known as a blind reconstruction, since no prior information was input to the inversion algorithm (Fig. 2a, f). From these blind results, it can be observed that the overall structure of the leg has been estimated; nevertheless, the fibula has not been detected and the tibia bone was poorly reconstructed in the real part of the relative complex permittivity.

Thus, several image enhancement procedures were followed (detailed in Sect. 5.2) such as incorporating prior information about the tissues’ dielectric properties and leg’s structure (Fig. 2b, g), changing the imaging domain to fit the model area (Fig. 2c, h), relocating the antennas around the leg (Fig. 2d, i), and changing the matching medium (AquaSonic 100 ultrasound gel) (Fig. 2e, j). These enhancements resulted in improved reconstruction results.

Furthermore, Fig. 9a shows the new locations of the imaging domain and antennas with respect to their old positions and the outermost contour of the leg.

In addition, to be able to use AquaSonic 100 as a matching medium, its electrical properties had to be known (details in Sect. 5.2.4). This was not found in the literature, so it was measured experimentally using Keysight’s N1501A Coaxial Dielectric Probe [44]; the results of the measurements between 0.5 GHz and 1.5 GHz are shown in Fig. 9b. Furthermore, the value of the gel’s relative complex permittivity at 0.8 GHz is $\epsilon _{r} = 71.4 - j 10.3$.

It is should be noted that the mid-values of the real part relative permittivity used for the prior information when using the glycerin/water solution as a matching medium versus using the ultrasound gel are 26 and 33, respectively. Both values are close to each other and in the range between the highest and lowest permittivity values of the tissues in the leg model. Thus, the successful reconstructions using both matching mediums as part of the prior information show the potential of utilizing ultrasound gels for MWT applications relative to the conventional glycerin/water.

From the reconstructions shown in Fig. 2, it can be seen that the estimation of tissue dielectric values was enhanced gradually with each enhancement scenario. Furthermore, the utilization of an ultrasound gel gave comparable results to the glycerin/water mixture. Despite the latter being very effective for breast microwave imaging [45], it is a liquid and will not be suitable for wearable MWT applications.

### Prior information extraction

In the previous section, prior information about the structure of the model was determined from the same numerical MRI-based model used in the forward problem to prove the concept of image enhancement. However, in a real MWT system, the numerical model is not available. Such information can normally be obtained either by a trained eye experienced in medical imaging such as in [46], or using multiple imaging modalities like ultrasound imaging [47] and MRI [48]. Herein, we utilized the blindly reconstructed image to obtain information about the structure of different tissues. The complete procedure is briefly described in Sect. 5.3 using the k-mean clustering algorithm. This algorithm clustered or grouped different tissue layers according to their relative permittivity and conductivity values. Thus, such clusters were used to determine tissue boundaries prior to using them within the inversion algorithm with the same enhancement steps mentioned in the previous section.

**Fig. 3:**
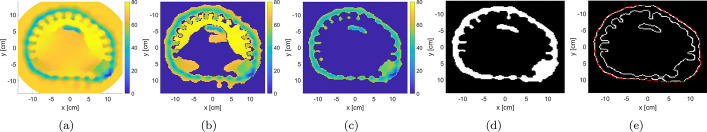
Structural prior information extraction procedure from the real part of the blind reconstructions: **a** blind inversion reconstruction; **b** matching medium removal; **c** low-permittivity cluster extraction; **d** smoothed binary image; **e** boundary formation using edge detection

The results of the clustering algorithm are represented in Fig. 3. The figure shows the algorithm when applied on the real part of the relative complex permittivity blind reconstruction (Fig. 3a), while using the ultrasound gel as the matching medium. The image goes initially into a pre-processing step to discard the matching medium values (Fig. 3b). Furthermore, the clustering algorithm is successfully divided the image into two regions, high permittivity and low permittivity. Furthermore, the low-permittivity region outlined the structure of the fat and the leg in general (Fig. 3c). Therefore, it was used for detecting the edges and the outermost boundary of the model (Fig. 3d, e). The figure shows successful reassignment of the pixels that are related to the outermost layer, but with the additional extraction of some pixels within the OI. This is considered acceptable as the main objective of the whole procedure is to estimate the boundaries of the layers regardless of the values within them.

**Fig. 4:**
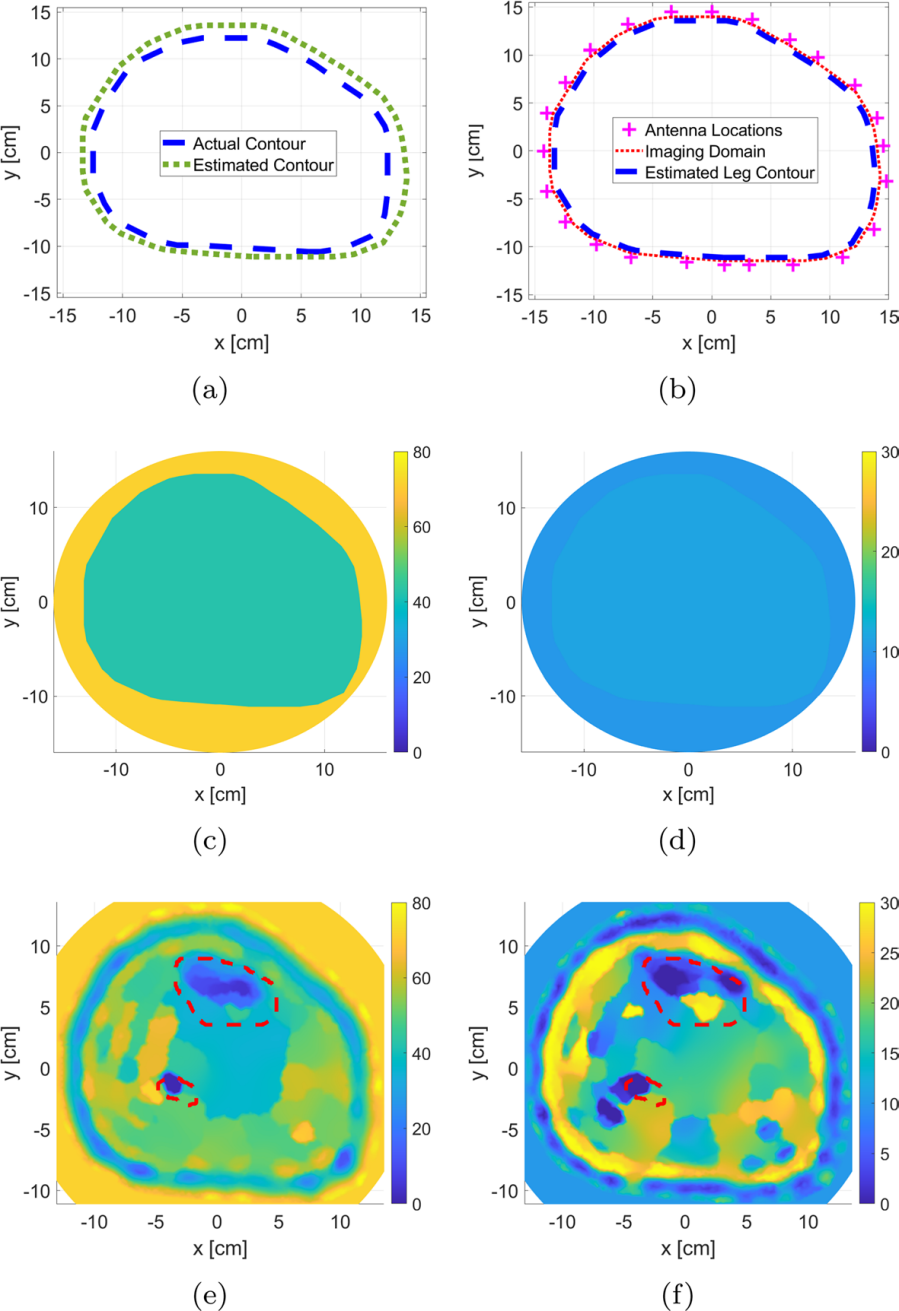
The use of the extracted prior information using the k-mean clustering algorithm applied to the blind MWT image: **a** comparison between the original and estimated leg contour; **b** imaging domain and antennas locations with respect to estimated outermost leg boundary; **c**, **d** real and imaginary components of the inhomogeneous background relative permittivity; **e**, **f** real and imaginary components of inversion algorithm relative permittivity reconstruction. The red-dotted lines indicate the bones’ actual locations

The procedure for estimating the leg’s contour through k-mean algorithm and using it as prior information is shown in Fig. 4a, b. The procedure for estimating the leg’s contour through k-mean algorithm and using it as prior information is shown in Fig. 4. The calculated $L_2-$norm for the difference between the actual boundaries versus the estimated one was $15.6\%$; this indicates that the procedure is successful in estimating the leg’s contour with very low error (Fig. 4a). Furthermore, the location of the imaging domain and antennas relative to the estimated boundary are shown in Fig. 4b.

The extracted prior information is incorporated as inhomogeneous background is shown in Fig. 4c, d. Finally, the results of the inversion algorithm using the extracted prior information are shown in Fig. 4e, f with the actual bones’ locations depicted in red-dotted lines. For the FEM-CSI results, it is clear that the various layers of the leg have been estimated by the algorithm. Furthermore, the two bones are detected as can be observed from the real-part reconstruction result.

### Monitoring bone density variations

As discussed in the introduction, bones’ density is measured via the BVF parameter. Furthermore, changes in bones’ electrical properties are inversely proportional to BVF variations. Here, we monitored these variations in MWT reconstructed images using five different BVF scenarios. In each scenario, the bones’ electrical properties were estimated, as shown in Table 1.

**Table 1:** The selected bone volume fraction (BVF) scenarios and the corresponding relative permittivity values

BVF scenarios	Relative permittivity ($\epsilon _r$)
0.50	$13 - j 3.0$
0.45	$14 - j 3.05$
0.35	$16 - j 3.1$
0.25	$18 - j 3.2$
0.10	$23 - j 3.4$

These values were interpolated based on the study conducted in [27] on porcine trabecular bones tissues. We used this study as a base to our investigations on human cortical bones tissues (obtained from [49]). This study was used only to interpolate BVF values according to the relationship found between bone mass loss and relative permittivity values. Thus, bones with 0.5 BVF were selected as an example of healthy bone case. Moreover, bones with 0.1 BVF was a scenario with severe bone density loss. It is worth noting that in [27], authors have used a frequency of 0.9 GHz to image bones. The relationship found between bones and permittivity values was used to interpolate bone values changes on the 0.8 GHz frequency range used in the proposed study.

**Fig. 5:**
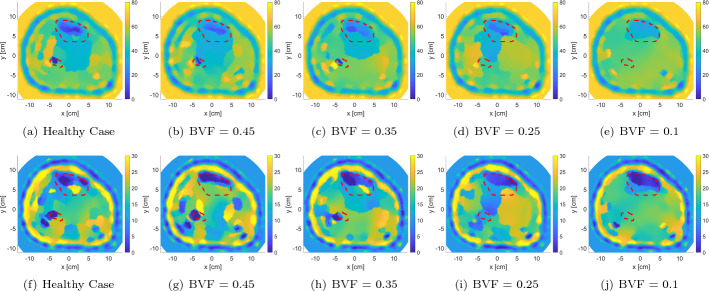
Enhanced relative complex permittivity reconstructions of the leg model with varying BVFs using prior information extracted from blind inversion (top row: real part; bottom row: imaginary part). The red-dotted lines indicate the bones’ actual locations

The synthetic data set was initially inverted blindly with no prior information. The blind reconstruction results were used to extract prior information about the leg, as discussed in Sect. 2.2. This prior information was used to adjust the inversion algorithm parameters as described in Sect. 2.1. After including the enhancements, FEM-CSI is run again. The results of the reconstructions are shown in Fig. 5 with the red-dotted line indicating the bone locations. As expected, the estimated bones’ relative permittivities change with varying BVFs. More specifically as the BVF is decreasing, the dielectric constant of the tibia bone is increasing, while the fibula bone is shrinking in size and eventually becoming undetected. Furthermore, it is worth to be mentioned that when bones are healthy, that is, 0.5 BVF, the algorithm tends to under-estimate the reconstruction of bones, whereas when bones are in severe conditions (0.1 BVF), the algorithm over-estimates bones values closer to the surrounding muscle tissues.

**Fig. 6:**
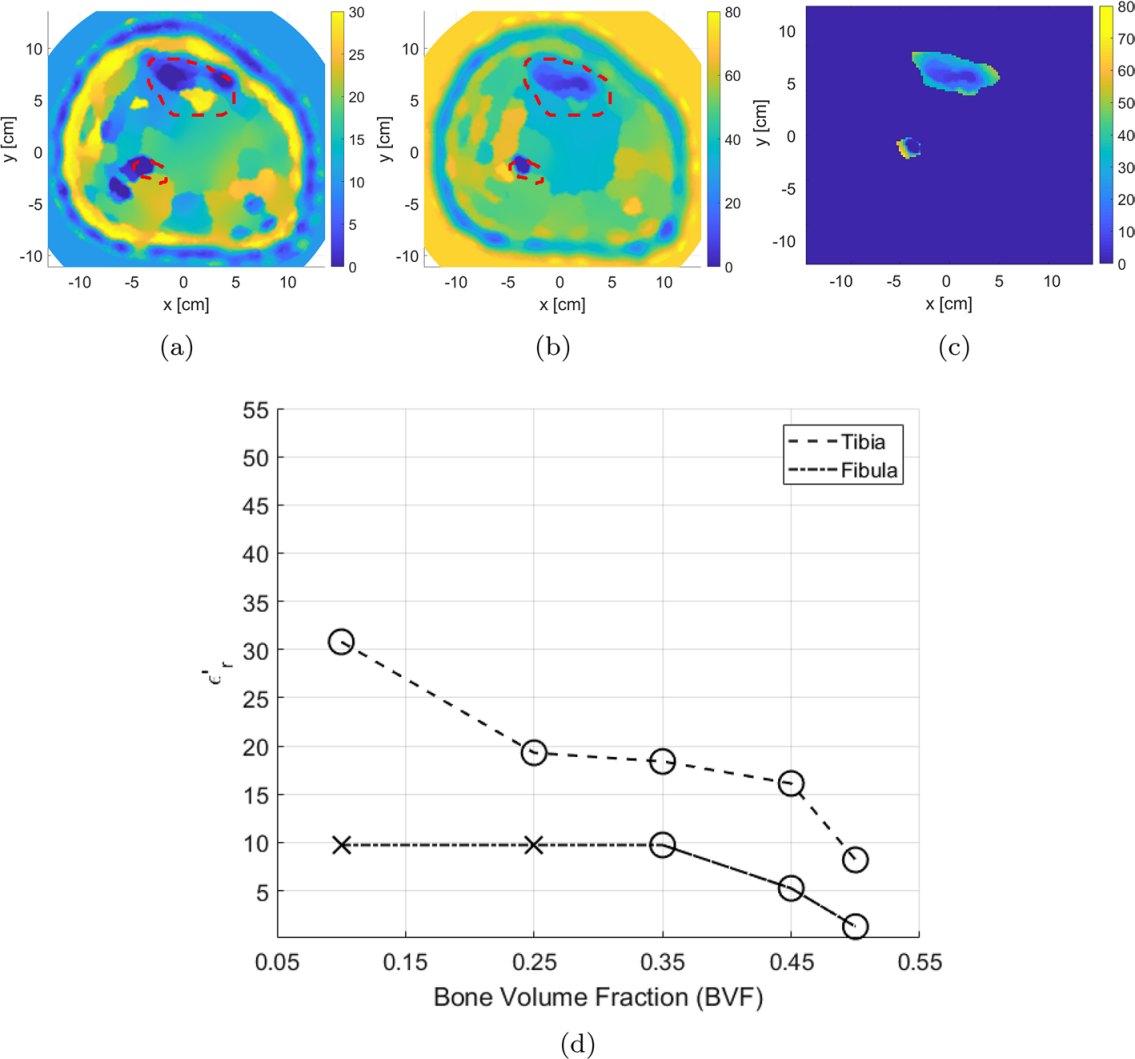
The model with 0.5 BVF: The process for calculating the mean of the reconstructed bones’ dielectric constant using an expert-eye localization technique: **a** imaginary-part localization; **b** real-part masking; and **c** extracted segments; **d** line plot representation of the mean value of the real part relative permittivity of extracted bones

To analyze changes in bones, an algorithm referred to as expert-eye was developed to extract their electrical properties from the inversion results (described in Sect. 5.4). The complete process for calculating the mean of reconstructed bones’ dielectric constant is demonstrated in Fig. 6 for 0.5 BVF scenario. It should be noted that the permittivity values for the bigger tibia bone were always estimated as this bone was visible in all these reconstructions. On the other hand, the smaller fibula bone was not reconstructed always by the inversion algorithm, and thus, its permittivity value was not estimated in all scenarios. Nevertheless, since the tibia was always estimated, this bone can be used solely to detect variations in bones’ permittivities for different BVF.

The procedure to calculate the bones’ mean dielectric constant was applied to the leg model images, where values for each of the five BVF scenarios were obtained. The results are presented in Fig. 6d. The BVF versus the calculated mean for the bones’ real part of the relative complex permittivity is plotted. Furthermore, there are two lines representing the tibia bone and fibula bone. For each line, if the inversion algorithm is successful in reconstructing the bone, the marker is represented with a circle ($\circ$); otherwise, failure is represented with a cross ($\times$). The line plot of bone permittivity values shows clearly that as BVF increases, the dielectric constant decreases for the tibia bone; this means that MWT, with all the suggested enhancements, can be used to detect the degradation of bones’ health.

## Discussion

We developed an approach for a numerical MWT system that is capable of monitoring bone density variations according to changes made to bones during degradation or treatment. The advantage of having such system lies in the ability of utilizing a safe and non-ionizing radiations to detect variations in the relative complex permittivity and illustrate it as a 2D image. This proposed idea paves the way towards implementing a user-friendly real-life MWT system that can be wearable. Furthermore, it allows for taking multiple imaging sessions within short periods without causing any harm to the tissues being imaged. The evaluation of this proposed idea within a numerical study prior to any clinical testing sets the base to the realistic imaging scenario in terms of the feasibility and expected outcomes. In addition, it is essential to test the robustness of imaging algorithms and enhancement techniques for the use in experimental and clinical trials.

The developed finite-element model closely simulates an actual cross-sectional slice of the lower leg, as a total of 254,044 triangles supported by 127,153 nodes were used to build the model. This allows for treating each tissue layer as a bulk object, especially when dealing with the incident electric field and its scattering effect. Although the blind reconstructions did not locate both bones accurately within the image, it was enhanced through several enhancement procedures that simulated closely a configuration of a wearable MWT system (prior structural information about tissues, closer imaging domain and antennas location, and an ultrasound gel). However, in a real-life system, it is considered difficult to exactly estimate these information about the structure and boundaries. This requires an expert to try to build the outermost boundary of the OI being imaged, which results in different levels of error between multiple experts, that is, radiologists. Furthermore, an MRI system can be used; however, this does not allow for frequent monitoring as these systems are not cheap and feasible when it comes to taking weekly sessions. Thus, we proposed an approach that estimates these structures from a previous image (most likely the blind image) of the same OI within similar MWT configuration. The decision on the clustering algorithm (k-means) was taken to ensure minimal complexity in the approach. Although this method is considered simple in calculations, it provides efficient results for problems involving bulk regions within images. An MWT reconstructed image may contain random values within the bulk objects. Thus, as the main objective in this work was to extract the outermost structure, it had no issues as the lowest values usually lie in the fat region surrounding the leg contour. Such approach can be applicable for imaging other objects within the human body, that is, breast imaging, as the fat is localized in the surroundings of the OI.

Since the gold standards in evaluating bone density involve X-ray, which an an ionizing and harmful radiations, it is desired to have an imaging modality that is simple, yet effective, and does not cause any harm to tissues. The successful estimations of bones permittivity at different levels of BVF illustrates the feasibility of monitoring the density through the changes in dielectric properties of the bones. However, as BVF decreases, that is, reaching a severe case of osteoporosis, the smaller bone (fibula) starts to completely disappear from the reconstruction. This could be due to small nature in terms of bones tissue. Additionally, this can be correlated with the increase in the real part of the relative permittivity which makes it harder for the algorithm to successfully reconstruct its values.

### Limitations

Although three-dimensional (3D) microwave imaging provides richer information for analysis of tissues, the successful 2D MWT system structure proposed in the current study is considered less complex to model, time efficient in calculations, simpler in use, and less expensive when built as a wearable system for frequently usage by doctors and patients. Furthermore, optimizations of inverse algorithm using 3D imaging systems require extensive calculations, especially when considered with a framework of a deep or machine learning model, which is a future aspect for the current work. After illustrating the feasibility of utilizing 2D MWT for bone variation monitoring, an experimental study that utilizes anatomical phantoms will be considered in future to encounter all real environment factors that may face the proposed approach, which was not possible in the current study (being as a numerical study). In addition, clinical testing of a real-life case of osteoporosis monitoring can be considered while relying completely on the MWT system for reconstruction, enhancement, and observing variations in dielectric properties of bones during treatment. Finally, as the main future works for this study include the design of an actual MWT system that could be wearable, the size of antennas should be taken into consideration. The proposed 24 antennas may be hard to place around the leg, so further studies require testing the optimal numbers of antennas taking into consideration the reconstruction performance when antennas are reduced.

## Conclusions

In this paper, a full numerical study is presented on the feasibility of designing a MWT system for bone health density monitoring. The study is conducted within the framework of the finite-element contrast source inversion method (FEM-CSI). First, enhancements to a conventional MWT system are applied to improve the inversion algorithm results as well as enable the system to be wearable. Next, to apply the suggested enhancements, prior information about the leg is extracted using blind inversions. During the preceding two investigations, commercial medical ultrasound gel is considered as matching medium. In the last section, a comprehensive procedure is followed to determine the efficacy of the proposed techniques to detect variations in bone density. The conclusion is that an MWT system can be designed and used to monitor changes in bones’ health by estimating their dielectric constant. The future work beyond this paper will focus on the implementation of such an MWT system.

## Materials and methods

### Numerical model and the forward problem

#### Generating anatomically realistic model

**Fig. 7:**
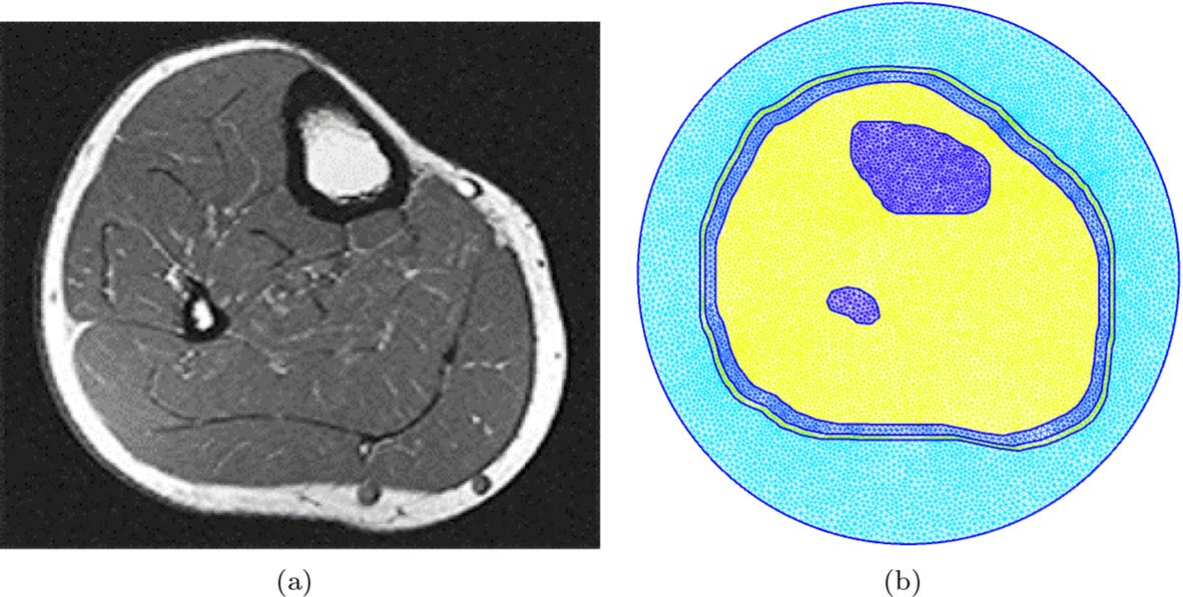
The cross-sectional MRI image and the corresponding finite-element model and mesh: **a** MRI image [50], and **b** finite-element model

The initial step of the study was to create the anatomically realistic model of human leg cross-section using MRI image. This model will be used to generate synthetic numerical data using an FEM forward solver. In this paper, the cross-sectional MRI image representing the middle-part of a human lower leg was selected from [50], as shown in Fig. 7. Next, the MRI image was imported into MATLAB, and the points corresponding to the boundaries of each tissue layer are extracted; these include skin, fat, muscle, tibia, and fibula boundary points. The bones considered for this work were from the cortical part of bones.

For generating the triangular mesh, the extracted boundary points were input to GMSH [51] and joined together to create the various tissues’ surfaces. Furthermore, the whole leg model was surrounded by a circle, which represents the boundary of the problem’s computational domain. When the geometrical model was ready, a 2D triangular mesh is generated, such that the characteristic length (which is the length of a mesh triangle’s side) is smaller than $\lambda /10$, where $\lambda$ is the wavelength calculated as

1 $$\begin{aligned} \lambda = c/\left( f \sqrt{\epsilon _{r,\text {max}}}\right) . \end{aligned}$$

Here, $c = 3\times 10^8$ [m/s] is the speed of light in free space, *f* is the simulation frequency, and $\epsilon _{r,\text {max}}$ is the largest possible value of the dielectric constant within the model. The finite-element meshes for each model are shown in Fig. 7. It should be noted that the characteristic length needs to be small enough to capture the details of the structure, as well as to have sufficient triangular elements to represent the various tissues in the leg.

#### Solving the forward problem

The objective of the forward problem was to simulate an MWT system when given information about the OI’s electrical properties, as well as the problem domain parameters. The simulation results were used to calculate the electric field values at the receiving antennas’ locations.

As mentioned earlier, for this study, the forward problem was solved using an in-house 2D FEM solver developed by Zakaria et al. [37]. The inputs for the solver are as follows:

- Model: The anatomically realistic 2D triangular mesh.
- Operating Frequency: 0.8 GHz.
- Transmitters’ (TX) and receivers’ (RX) configuration: 24 point-sources (TX) and 24 point-sinks (RX) collocated and equally distributed on a circle of radius 15 cm surrounding the model.
- Matching medium surrounding the OI: 80:20 glycerin/water solution with relative complex permittivity of $\epsilon _r = 26 - j 18$ [52, 53].
- Relative complex permittivity of various human tissues in the model: Skin ($42 - j 18.8$), fat ($11 - j 2.3$), muscles ($55 - j 20.5$), and healthy cortical bones ($13 - j 3.0$) [25, 49].

**Fig. 8:**
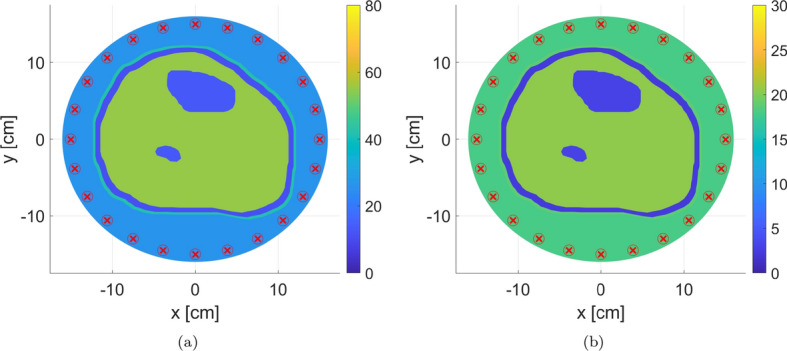
The real and imaginary parts of the relative complex permittivity at frequency of 0.8 GHz. The antenna locations are indicated by ($\times$) and ($\circ$) markers for transmitters and receivers, respectively; the transmitters and receivers are collocated. Tissues relative permittivity values were obtained from [25, 49]

It is worth noting that human cortical bones’ relative permittivity values were interpolated based on [49] for the frequency selected in this study. As an example, the real and imaginary parts of the relative complex permittivity for various mesh regions are shown in Fig. 8; in each figure, the TX and RX locations are indicated by ($\times$) and ($\circ$) markers, respectively.

It should be noted that in literature, the simulation frequency for biomedical MWT applications ranges from 0.5 to 3 GHz [2]. In our work, the frequency of 0.8 GHz was selected to ensure enough penetration for electromagnetic signals occurred inside the leg (the larger the frequency, the smaller is the signal penetration and the higher are the propagation losses), while at the same time, taking into consideration that any antenna built for the MWT system should be reasonably size (the smaller the frequency, the larger the antenna).

As for number of transmitters and receivers, 24 antennas were chosen (acting as transceivers) as they will be enough to capture the scattering effect from different locations around the leg, while at the same time not providing redundant information to the inversion algorithm [54, 55]. Each antenna acts as a transmitter for the electromagnetic signals, while at the same time, it acts as a receiver for the scattered signals because of the existence of the OI.

Finally, for the matching medium, the 80:20 glycerin/water solution mixture proposed by Meaney et al. [45] for human microwave imaging was used as a starting point for the choice of an immersion medium. Later, in this paper, another matching medium readily available in the medical field is chosen.

### Blind reconstructions and inversion enhancements

After collecting the synthetic data for developed model, these data were used as input for the inversion algorithm. The inversion algorithm considered in this work is the multiplicatively regularized contrast source inversion (CSI) method implemented within the framework of the finite-element method (FEM) [36, 38]. The details of the FEM-CSI algorithm as well as multiplicative regularization are outlined in [37].

To prevent inverse crime, the forward and inversion meshes were chosen to be different. In addition, $3\%$ uniform noise was added to the scattered field synthetic data as done in [36, 56]; this is a common practice in numerical microwave imaging studies. Furthermore, the imaging domain, where the electrical properties of the OI are to be estimated, was chosen to be a circular domain with a 14 cm radius centered in middle of the problem domain. The inversion algorithm was allowed to run for 1000 iterations to compare the imaging performance consistently; by the 1000th iteration, the CSI algorithm converges for all performed inversions. It should be noted that a recent study on the stopping criteria for algorithms used in microwave imaging can be found in [57].

To enhance the image reconstruction, prior information (inhomogeneous background) about the human leg was incorporated. This information includes the contour of the leg and/or information about the bulk tissues, such as skin, fat, and muscles. The prior information was retrieved initially from the forward problem MRI-based model to prove the concept of image enhancement. However, the upcoming sections discuss an algorithm that was developed to extract this information from the blind reconstructions automatically.

#### Utilizing inhomogeneous background

For the blind inversions, the electrical properties of the empty imaging domain were homogeneous with the relative complex permittivity set to that of the matching medium. Nevertheless, as shown in [36, 46, 58, 59], by having an inhomogeneous background distribution related to the OI, the reconstruction results can be improved. Since the FEM-CSI algorithm is being utilized, incorporating prior information as inhomogeneous background can be readily done.

In this work, the incorporated inhomogeneous background is the outer boundary of the human leg, with the enclosed surface by the contour assigned a relative complex permittivity value of

2 $$\begin{aligned} \epsilon _{r,{\text {mid}}} = \left( \epsilon _{r,{\text {high}}}+\epsilon _{r,{\text {low}}}\right) /2. \end{aligned}$$

Here, $\epsilon _{r,{\text {high}}}$ and $\epsilon _{r,{\text {low}}}$ are, respectively, the highest and lowest values of expected bulk permittivity within the imaging domain. In the example herein, the highest value corresponds to the muscle’s relative permittivity while the lowest value is the fat’s relative permittivity.

Unlike the work done by [46, 60], no information about the different tissues layers within the human leg was incorporated. The whole bulk of the leg was assigned the same relative complex permittivity of $\epsilon _{r,{\text {mid}}}$. From a practical point view, this is advantageous as extracting the different tissue layers inside the leg, although achievable, is complex.

#### Changing the imaging domain

In the blind inversion, the imaging domain was circular with a radius of 14 cm. This results in having the surrounding matching medium electrical properties calculations within the inversion domain. Thus, changing the imaging domain to exactly fit the OI contour helps in reducing the number of unknowns within the reconstruction area and thus the computational complexity. As demonstrated by [12, 18], this type of *conformal* imaging domain improves the reconstruction results. Herein, the imaging domain is selected to span exactly the outer boundary of the human leg as obtained from the MRI-based leg model.

#### Relocating antennas

By having the antennas at a close distance from the OI, the loss of the transmitted or received signals to the matching medium is reduced as more EM energy is directed towards the OI itself or captured by the receiving antennas. Theoretically, this should improve the reconstruction results in MWT. Changing the antennas’ locations was previously discussed by [47] for localizing tumors using an MWT system designed for breast cancer detection. The locations of the antennas were adjusted to surround the contour of a breast using a secondary ultrasound system. The results showed that by having the antennas located in a close distance from the OI, the tumor localization improves, in comparison to having the antennas’ on a circular surface.

In this study, the location of antennas was changed from being on a circle of 15 cm radius to antenna locations conforming with the OI’s outermost contour. This would simulate a wearable MWT system scenario. To prevent the inclusion of antennas within the imaging domain, the antennas’ measurement surface was extruded slightly beyond the OI model’s outermost contour. After changing the antenna positions, synthetic data are collected again.

**Fig. 9:**
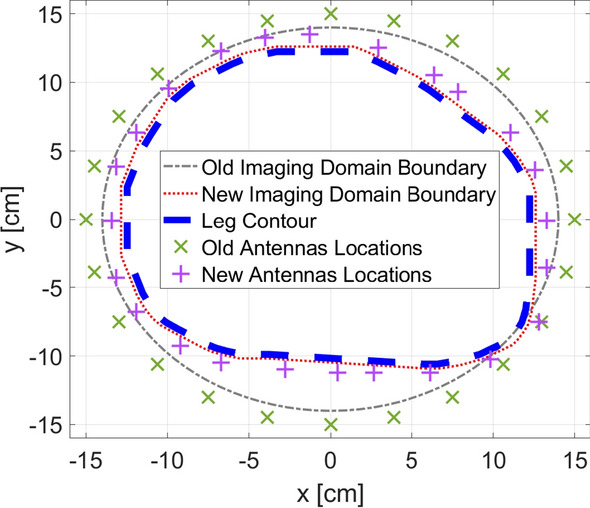
New locations of imaging domain and antennas with respect to the old positions and the contour of the leg

Furthermore, Fig. 9 shows the new locations of imaging domain and antennas with respect to their old positions and the outermost contour of the leg.

#### Changing the matching medium

As mentioned earlier, the matching medium selected for the previous simulations was a mixture of glycerin and water. This matching medium was used as a starting point as it was very effective for breast microwave imaging [45] as it reduced the reflections at the matching medium/skin interface. However, a disadvantage of this mixture solution is that it is liquid and will not be suitable for wearable MWT applications.

An alternative matching medium that is considered in this work is the ultrasound gel, which is commonly used for medical imaging purposes. The ultrasound gel is aqueous, bacterio-static, non-sensitizing, and non-irritating. Furthermore, since it is of a gel form, it can be easily applied and cleaned in a wearable MWT system. The world standard for ultrasound gels is the AquaSonic 100 [61]. To the best of the authors’ knowledge, there has been no previous research on the use of AquaSonic 100 ultrasound gels in microwave tomographic applications; thus, it is considered investigated as a matching medium in this section.

**Fig. 10:**
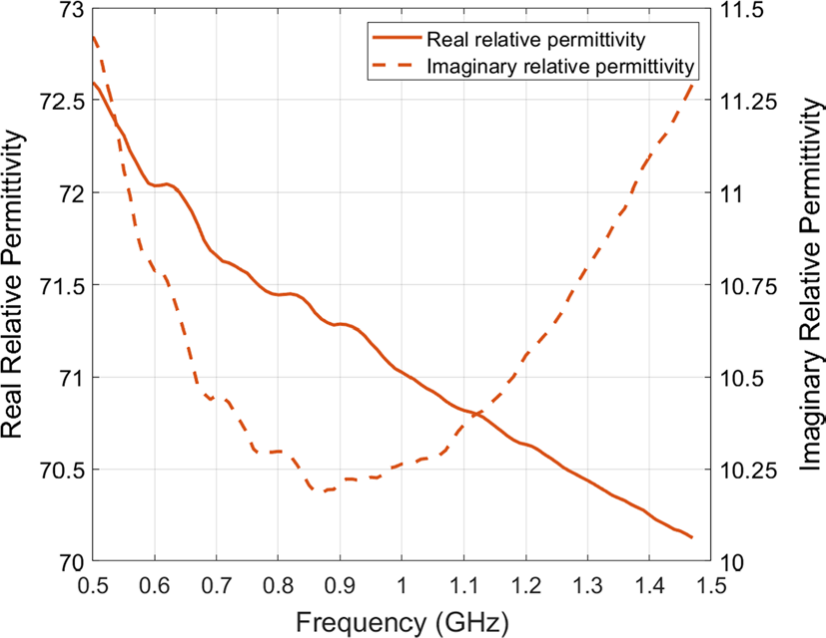
Relative complex permittivity value of AquaSonic 100 gel

To be able to use AquaSonic 100 as a matching medium, its electrical properties need to be known. This was not found in the literature, so it was measured experimentally using Keysight’s N1501A Coaxial Dielectric Probe [44]; the results of the measurements between 0.5 GHz and 1.5 GHz are shown in Fig. 10. Furthermore, the value of the gel’s relative complex permittivity at 0.8 GHz is $\epsilon _{r} = 71.4 - j 10.3$.

With the new matching medium, noisy synthetic data were generated again. Next, the inversion algorithm was run using the leg’s prior information to incorporate an inhomogeneous background, change the imaging domain, and relocate the antennas as discussed in the previous sections. The results of the reconstructions are shown in Fig. 2e, j. It can observed that the two bones have been detected by the algorithm along with the different bulk regions of the leg. Therefore, using the ultrasound gel as a matching medium is applicable in biomedical MWT; thus, it will adopted for the remainder of the work in this study.

### Prior information from k-means algorithm

In the previous section, prior information was obtained from the MRI-based leg model; however, in a real MWT configuration, this model is not available. Therefore, we have used an image processing algorithm (*k*-means) [62] to extract structural information about the OI using the blind reconstructions. In this technique, the selected image is iteratively segmented into a certain number of clusters determined by the user. Each cluster includes all values within the image that are close to a calculated centroid [63].

#### Pre-processing

The utilized *k*-means clustering algorithm applies on a uniform square grid; however, in FEM-CSI, the estimated electrical properties are calculated on the nodes of the triangular mesh. Thus, the first step in pre-processing was to interpolate the inversion algorithm output onto a uniform square grid. In an actual MWT system, this step can be omitted due to not having numerical models but rather a direct 2D image. After obtaining a square grid for the relative complex permittivity of the blind reconstructions, pixels with values close to the matching medium electrical properties were extracted and assigned a value of one for later further processing. The purpose of this step is to ensure that the *k*-means algorithm is applied only on pixels corresponding to the reconstructed OI.

Since the estimated values of the reconstructed images are non-uniform, and may be an over- or under-estimate of the actual matching medium relative complex permittivity, the extracted pixels are selected, such that the real part of the relative permittivity is within the following limit:

3 $$\begin{aligned} \left( \epsilon '_{r,\text {match}}-4\right) \le \epsilon '_{r,\text {reconstr.}} \le \left( \epsilon '_{r, \text {match}}+4\right) . \end{aligned}$$

Here $\epsilon '_{r,\text {match}}$ and $\epsilon '_{r, \text {reconstr.}}$ are the real parts of the relative complex permittivity for the matching medium and reconstructed images, respectively. The selection of this limit was determined in an ad-hoc manner; nevertheless, the selected criteria worked for every tested matching medium (glycerin mixture or ultrasound gel). Several trial-and-error tests were performed to decide on the limit boundaries. The limit was successful in removing matching medium values within the range of $\pm 4$. Using the ultrasound gel as a matching medium, the limit in Eq. 3 is $67.4 \le \epsilon _{r}^{'} \le 75.4$. Again, after the background pixels are identified, they are assigned a value of one.

#### *k*-means clustering algorithm

The pre-processing yielded primarily of two regions, which can be referred to as Low-Permittivity and High-Permittivity regions. The Low-Permittivity region has relative permittivity values close to that of fat and bones, while the High-Permittivity region has values close to that of muscle and skin tissues. The *k*-means clustering algorithm was applied on this image to extract these two clusters. After applying the algorithm, two regions are extracted with their centroids calculated to be 46.10 and 67.55 for the Low-Permittivity and High-Permittivity regions, respectively. From the two extracted regions, the Low-Permittivity cluster is retained, while the High-Permittivity region is omitted. This is done, since the Low-Permittivity region, representing the fat tissue, is enough to estimate the contour of the leg.

#### Outermost boundary extraction

To build the fat’s contour from the outermost boundaries of the Low-Permittivity cluster segment, a binary image of the cluster was initially generated. The resultant binary image will include mostly a big connected portion that corresponds to the fat tissue, along with smaller clusters scattered around it; these smaller clusters are smoothed out and removed.

Next, an edge detection algorithm was applied on the remaining segments of the smoothed binary image to get the outermost boundaries of the fat cluster. In this algorithm, the Canny technique was selected [64]. The output of this process is the (*x*, *y*) coordinates of the pixels representing the outermost edge of the fat cluster.

#### Structural model formation

Using the (*x*, *y*) coordinates extracted in the previous step, an estimated model of the OI was built. Since the extracted points represent the boundary of fat tissues, the skin contour (which represents the actual contour of the leg) was formed by slightly extruding the points 4.5 mm outwards, which is a rough estimate of the skin thickness in human [65].

Next, the extruded points were exported to GMSH to build the prior information leg model, and then generate the triangular mesh used in the inversion process. The prior information was incorporated as inhomogeneous background in FEM-CSI and was used to change the imaging domain shape and relocate the antennas.

### Expert-eye bone localization

The main goal of the expert-eye localization was to extract bones dielectric properties from the reconstructed MWT images. Thus, monitor the variations in bone density as reflected by the relative complex permittivity. The first step of the algorithm is to manually locate bones’ regions-of-interest (ROIs) in the imaginary-part reconstructed image. These are regions where the imaginary part of the relative permittivity is low and where the bones are expected to be. This is referred to as expert-eye localization, as a radiologist looking at the MWT reconstructed image can distinguish the bones from the surrounding tissues with ease. Next, the masks of these ROIs are applied on the real part of the reconstruction to extract the dielectric constant values within the regions. Since these masks may contain muscle tissues surrounding the bones, values within the regions that are more than the first quartile of the ROIs’ mean dielectric constant are discarded. Finally, the mean of the remaining values is calculated and presented as an estimation of the bones’ real relative permittivity.

## Data Availability

The data used to support the findings of this study are available from the corresponding author upon request.

## Abbreviations

MWT: Microwave tomography
MRI: Magnetic resonance imaging
FEM-CSI: Finite-element method-contrast source inversion
TM: Transverse magnetic
OI: Object-of-interest
BVF: Bone volume fraction
